# A Narrative Review of Burnout Syndrome in Medical Personnel

**DOI:** 10.3390/diagnostics14171971

**Published:** 2024-09-06

**Authors:** Andreea-Petra Ungur, Maria Bârsan, Andreea-Iulia Socaciu, Armand Gabriel Râjnoveanu, Răzvan Ionuț, Letiția Goia, Lucia Maria Procopciuc

**Affiliations:** 1 Department of Occupational Medicine, Iuliu Hatieganu University of Medicine and Pharmacy, 400012 Cluj-Napoca, Romania; andreea.ladaru@umfcluj.ro (A.-P.U.); andreea.socaciu@umfcluj.ro (A.-I.S.); armand.rajnoveanu@umfcluj.ro (A.G.R.); ionut.razvan@umfcluj.ro (R.I.); 2 Department of Modern Languages, Iuliu Hatieganu University of Medicine and Pharmacy, 400349 Cluj-Napoca, Romania; letitia.goia@umfcluj.ro; 3 Department of Molecular Sciences, Medical Biochemistry, Iuliu Hatieganu University of Medicine and Pharmacy, 400012 Cluj-Napoca, Romania; luciamariaprocopciuc@yahoo.com

**Keywords:** burnout, occupational stress, healthcare workers, biomarkers

## Abstract

Burnout among healthcare workers has been extensively studied since its initial recognition in 1960, with its defining characteristics established by Maslach in 1982. The syndrome, characterized by emotional exhaustion, depersonalization, and low personal accomplishment, is exacerbated by work-related stress and has profound implications for individual and societal well-being. Methods: A review of the literature, including PubMed searches and analyses of risk factors and protective measures, was conducted to assess the prevalence, impacts, and biomarkers associated with burnout among healthcare workers. Various instruments for evaluating burnout were examined, including the widely used Maslach Burnout Inventory, alongside specific tools tailored to different occupational populations. Results: Healthcare workers, particularly physicians, exhibit significantly higher rates of burnout compared to the general population. Factors such as night shifts, workload, and exposure to biohazards contribute to elevated burnout risk. Biomarkers like cortisol, melatonin, and thyroid hormones have been linked to burnout, highlighting physiological implications. Conclusions: Burnout poses significant challenges to healthcare systems globally, impacting patient care, worker retention, and overall well-being. Identifying and addressing risk factors while promoting protective factors such as resilience and social support are crucial in mitigating burnout. Further research into prevention strategies and biomarker monitoring is warranted to support the mental and physical health of healthcare workers.

## 1. Introduction

In the context of healthcare workers, the notion of burnout first appeared in 1960 [1]. Freudenberger made the first observations about the concept in 1974, studying a group of employees from a free clinic, including himself [2]. He attempted a definition and described the physical and behavioural signs, the population at risk, but also suggested preventive measures and means to help those affected by it. The final definition of burnout was provided in 1982 by Maslach [3] as a syndrome with three dimensions: emotional exhaustion, depersonalization, and low personal accomplishment [4]. The JD-R (job demands-resources) model suggests that burnout develops through two key processes [5]. First, extreme job demands result in constant overtaxing, ultimately leading to exhaustion. Second, insufficient resources make it difficult to meet these job demands, causing withdrawal behaviour. This withdrawal eventually leads to disengagement from work. It can be argued that the interaction between job demands and resources is crucial for the development of burnout, encompassing both exhaustion and disengagement. By some authors, burnout is considered poorly managed work-related stress that can lead to decreased efficiency at the workplace and lower overall motivation and personal satisfaction, which are experienced by both the individual and the society [6].

**Figure 1:**
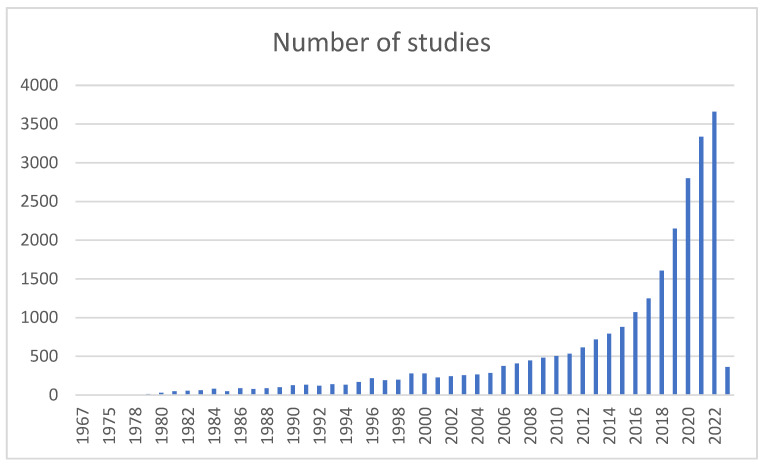
PubMed search results for “burnout” between 1967 and January 2023.

In seeing how mental, physical, and psychological health have since then been closely linked to our occupational activity, the phenomenon of burnout has become a widely discussed and observed subject [3]. A standard PubMed search using the terms “occupational stress” and “burnout” generates over 27,000 results from all over the world for the last 5 years alone, demonstrating a strong and steady ascendent trend (Figure 1) since the first study published in 1967.

Compared to the general population, physicians are more likely to record significantly higher burnout scores, as concluded by a study performed in the USA in 2012 [7], where one in two doctors was affected by burnout. The results were confirmed by the follow-up research conducted by the same team of researchers 7 years later [8]. Another team of researchers stated that medical personnel have the highest prevalence of burnout out of any other type of work [9]. The reasons for these discrepancies, when compared to the general population, could be linked to fast-paced work tasks, working night shifts, or exposure to biological and chemical hazards in the workplace [10,11].

Exposure to all kinds of biohazards occurs when dealing with patients, when handling or taking samples, and is a very common situation for all healthcare workers [10]. WHO estimated that over 3 million healthcare workers are injured by needles or sharp instruments, and a study conducted in Italy stated that approximately 80% of all cases with exposure to biohazards resulted from such healthcare accidents [12]. Such events can have serious financial and time-management consequences, implying the need for an epidemiological investigation, treatment, and medical care with possible long-term effects. Research conducted over 5 years showed nurses to be most often affected [13].

Shift work is commonly encountered in medical training, the medical profession, and patient care. Research has identified cognitive impairment in sleep-deprived physicians and changes in their electroencephalograms recorded at the time of and following the night shift [10].

Personnel active in the medical environment, especially in hospital settings, feel a higher demand and are more prone to showing signs of burnout [6]. A large study on US-active physicians identified important signs of burnout, depression, and even suicidal ideation, with specialities known for their workload and stressful environment (e.g., emergency medicine) being linked with higher rates [7].

The exposure to the new Coronavirus brought challenges for all kinds of employees, but medical personnel were most affected [14]. The COVID-19 pandemic was declared by WHO on 11 March 2020. This severe situation implied the implementation of new measures and rules in a short time, causing additional psychological burden for most employees, burnout being a possible consequence [15]. By the end of October 2020, the toll of infected people rose to 45 million and the death toll reached 1.2 million worldwide [16], with healthcare workers having a seven-times higher risk of infection [17,18]. The most affected by burnout were the medical staff in critical care and emergency departments [17].

## 2. Risk Factors for Burnout

**Figure 2:**
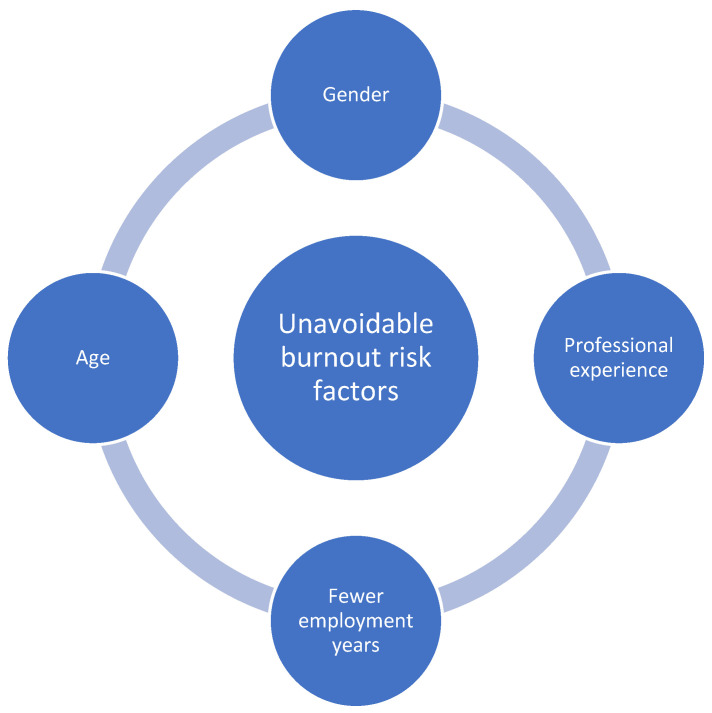
Unavoidable risk factors for burnout.

The first step toward preventing the occurrence of burnout in any population of workers is identifying the risk factors. This could be of use for the employee, as well as for the employer. Employees could positively influence risk factors, activities, and behaviours that can be controlled, whereas employers could lower stress levels at the workplace and increase job retention with the help of new business models. A systematic review conducted in 2022 classifies the risk factors into avoidable and unavoidable [19], with the latter representing internal or unchangeable features of the individual (Figure 2).

**Figure 3:**
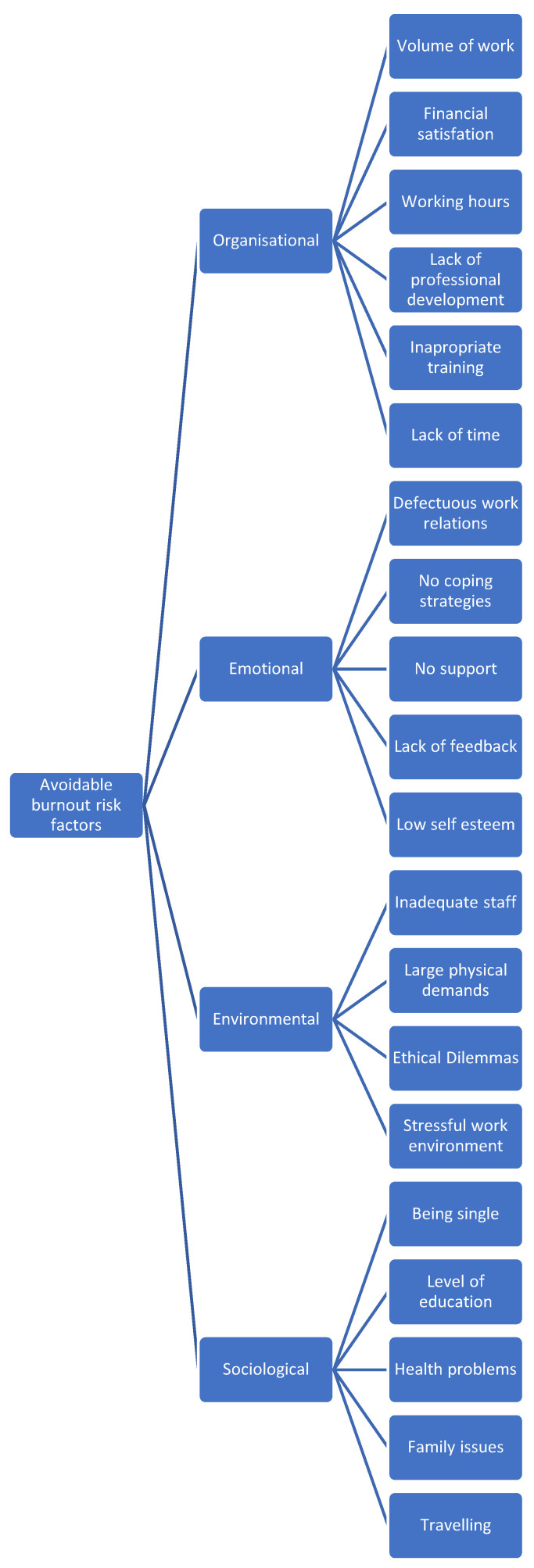
Avoidable risk factors for burnout.

The avoidable risk factors are modifiable aspects that an individual or an institution can regulate. Based on previous research, the authors of review [20], classified these risk factors further after a thorough analysis into one of four categories—see Figure 3.

A complex systematic review classified the risk factors for burnout in medical personnel (Table 1), as occupational and non-occupational [18].

## 3. Instruments Used for Evaluating Burnout

Generic-use instruments do not take into consideration the individual’s occupation and can evaluate the burnout levels in a wider range of occupational domains. On the other hand, specific instruments refer to an explicit occupation or domain, highlighting the harmful potential of different job-related activities with targeted questions. A description of the tools used for evaluating medical personnel can be found in Table 2. Each instrument has several dimensions with a specific number of items and is available in English (EN), or other languages.

Other questionnaires aimed at non-medical populations are the Shirom-Melamed Burnout Questionnaire [30,31], aimed at students and athletes; School Burnout Inventory [32,33,34], applied on students; Parental Burnout Inventory [35], evaluating parents; Teacher Burnout Questionnaire [36], used for teachers; and Burnout Questionnaire for Athletes [37], valid only for this group.

From the instruments mentioned above, the Maslach Burnout Inventory [3] is a questionnaire widely used to evaluate the impact of occupational stress due to work overload and poor management of the job’s requirements, which can be perceived as chronic stress. It has different versions, depending on the targeted population, and it evaluates the following three dimensions of burnout [3,38]:

- Emotional exhaustion (implies that the demands at the workplace are very high for the employee and the individual does not have enough emotional resources to manage stress);
- Depersonalization (the individual adopts a distant or even cynical attitude toward their colleagues);
- Low personal accomplishment (a consequence of the individual perceiving themself as less efficient and not being able to perform the job demands [3,39]).

Besides the characterization of the burnout phenomenon using the three previously described dimensions, additional differences between individuals can be illustrated using the following subtypes scales:

- The frenetic subtype: the employee has higher levels of involvement and motivation regarding the job demands, willing to make significant efforts to achieve goals, sometimes neglecting their personal life and health, usually found in highly demanding types of work and prone to exhaustion due to resource consumption [40,41];
- The underchallenged subtype: due to repetitive tasks and a monotonous job with low personal accomplishment, the employee tends to become bored, desires to change job, is often distracted, and has a cynical attitude toward work [24,25];
- The worn-out subtype: the employee is less dedicated, has no control over the job requirements, cannot recognize achievements, and is expected to neglect some of the tasks, with feelings of guilt and incompetence [24,25].

These subtypes can also be viewed as stages to pass through to eventual burnout: starting with the increased involvement of the frenetic subtype, continuing with exhaustion due to the difficulty of keeping up with increasing tasks, morphing into the underchallenged subtype, an individual distancing themself from the job to cope, and culminating with the worn-out subtype—demotivated, feeling less efficient at work [42].

## 4. Biomarkers Monitoring Burnout

When faced with an event able to produce acute stress, the main systems involved in the response reaction are the autonomic nervous system, the hypothalamic–pituitary–adrenal axis, and the hypothalamic–pituitary–thyroid axis [43,44]. Their actions lead to certain changes in hormone levels; increase the heart rate, blood pressure, and immune suppression; and intensify catabolic processes. Studies monitoring the effect of burnout mention several hormones connected with a high level of stress, among them are the following: melatonin, cortisol, DHEA-s, ACTH, thyroid hormones, and prolactin (see Table 3).

Melatonin, a molecule regulator hormone found both in mammals [47] and in plants [48] is a derivative of serotonin, produced by the pineal gland; its synthesis depends on well-functioning beta adrenergic receptors. The release of melatonin is correlated with changes in light, a biological clock controlled by the suprachiasmatic nuclei, activated by darkness, and depressed by light [49]. It influences many physiological functions and decreases with age and in various conditions, like neurological disease (e.g., Alzheimer’s), metabolic disorders (e.g., type 2 diabetes), cardiovascular disease, cancer, endocrine disease, and high exposure to stress [50]. Once darkness sets in, melatonin synthesis rises, and the indole hormone is released from the pineal gland into the blood stream and cerebrospinal fluid, its concentration being thus higher at night. This sends a very important circadian message for the normal functioning of the human body. A decrease in melatonin is expected when exposure to light during nighttime occurs. The variation in this hormone can be evaluated with the plasma or saliva melatonin level or by determining sulfatoxymelatonin from urine, which is the main hepatic metabolite [61].

The most important functions of melatonin are the ability to reduce oxidative stress through direct detoxification or indirectly by inhibiting the activity of prooxidative enzymes and stimulating antioxidant enzymes [61], acting as a physiological sleep regulator and controller for the sleep–wake cycle [49]. Melatonin promotes sleep, has beneficial effects in sleep disorders and chronic insomnia, and is found in patients of various ages suffering from depression, as well as attention deficiency and hyperactivity disorders [62], Alzheimer’s disease, dementia, and migraines [63]. Low levels of melatonin were identified in patients diagnosed with breast and prostate cancer [64]. Since occupational burnout syndrome has been described in association with insomnia [65], immune, inflammatory, and metabolic disfunctions, melatonin could prove useful not only in monitoring these conditions, but also in treating them.

The cortisol [52] level can be determined in saliva or blood before or after dexamethasone use. Prolonged exposure to stress causes lower values of cortisol and metabolites [38]. Due to circadian and diurnal differences in its secretion, cortisol has shown inconsistent results.

DHEA has a higher level [54] in young patients with less burnout, and it decreases in older subjects, especially in those who are stressed and score high in questionnaires for burnout.

Low levels of thyroid hormones and ACTH can be found in exhausted patients with prolonged exposure to stress, eventually inclined to develop burnout [47,55].

High levels of prolactin were correlated with a high burnout level in male patients, but not in female patients [56].

S100B was correlated with increased emotional exhaustion and depersonalization and might be used as a biomarker in patients with acute depression [57].

Higher levels of plasma BDNF [66] can be found in exhausted workers and patients with depression.

The proinflammatory cytokine release of TNFα [67] and IL-6 is likely to increase as a response to stress and increased burnout scores.

Increased levels of CRP-C reactive protein [68] and IL-6 were detected in patients dealing with high stress levels and depression.

A certain gene polymorphism of CRH-corticotropin-releasing hormone [52] is correlated with an increased level of burnout, emotional exhaustion being directly related to increased stress at work.

## 5. Protectors against Burnout

Different actions have been proven to protect against burnout (see Table 4). Studies have shown that there might be some protective factors [69] against burnout: grit, resilience, psychological flexibility, and benefiting from social support. Grit is defined as being perseverant, wanting something and doing everything possible to reach that goal [70]. Resilience is having the ability to take a step back from stressors and regain the necessary energy [69]. Psychological flexibility is one’s ability to focus and go on to reach the goal [71]. Having social support from one’s family, friends, and colleagues is a well-known protective factor likely to create a safety net strong enough to sustain the individual [72,73]. Enhancing social support has proven to be an effective method to reduce burnout. Other interventions are training programmes for developing skills in managing emotions in relationships with coworkers and patients.

Personality traits [74] like emotional intelligence and self-efficacy seem to have a positive role in preventing the occurrence of burnout. Emotional intelligence is understanding and coping with the individual emotions and feelings and relating this to job performances and demands. Self-efficacy is the ability to control the work environment and have better performances [75].

Earlier studies observed sociodemographic variables as burnout predictors or protectors: marital status, age, and sex [76]. A six-month study conducted in France just before the pandemic showed that general practitioners who were in a relationship were less likely to have an elevated level of burnout than single practitioners [77]. Another study conducted in Brazil [78] noted that single or divorced nurses who had a partner had a lower perception of workplace stressors, being less prone to developing burnout. Experienced female nurses tend to score lower in emotional exhaustion and depersonalization, meaning that more habituation at work might be a protector against stress [74]. Male nurses with neither family nor children reported higher scores of burnout [78].

The structure and level of evidence appear to be very relevant to the development of a successful intervention. Regular physical activity (both aerobic and strength) is an effective method for reducing burnout levels, especially in the dimensions of emotional exhaustion [79] and depersonalization, and is considered one of the best stress-coping mechanisms [80,81].

## 6. Repercussions of Burnout

Alcohol and/or drug consumption is one of the most common coping mechanisms used when dealing with stressful situations. Studies have shown a higher incidence of alcohol abuse in physicians with a high level of burnout, often associated with negative thoughts, lack of satisfaction at work, and low sense of accomplishment [82]. Drug abuse is often observed in workers complaining of high stress, emotional issues, increased workload, and prolonged work hours; 8 to 12 percent of medical staff develop this kind of addiction in their career [83].

According to some researchers, there are similarities between burnout and depression, both having the same causes and analogous symptoms [84]. They argue that there is insufficient evidence to consider burnout as a separate condition and suggest that the two primary dimensions of burnout (exhaustion and depersonalization) be viewed as depressive reactions to a stressful work environment. This relates to a significant scientific discussion regarding whether burnout syndrome should be regarded as a distinct illness with specific diagnostic criteria. The difference between burnout and depression is also underlined by multiple studies: burnout is typically described as a syndrome linked to work rather than a medical condition, while depression is a recognized disorder that has specific diagnostic criteria and can arise independently of context or in reaction to stressors.

Various thoughts and ideas prevent doctors from admitting their problem and seeking help, aggravated by the fear of losing their job, being stigmatized by coworkers, having professional relationships altered, being seen in a different light by the society, and being distinct from others, with all of the above leading to a vicious circle [85]. Individuals affected by depression have a high potential for developing suicidal ideas. In 2011, 1 in 16 doctors reported having thoughts of suicide, resulting from burnout [86]. The same study concluded that, among the physicians who experienced depression, 87.5% also reported symptoms of burnout. Additionally, 26.2% of those physicians exhibiting symptoms of burnout symptoms were also diagnosed with major depression [86].

**Figure 4:**
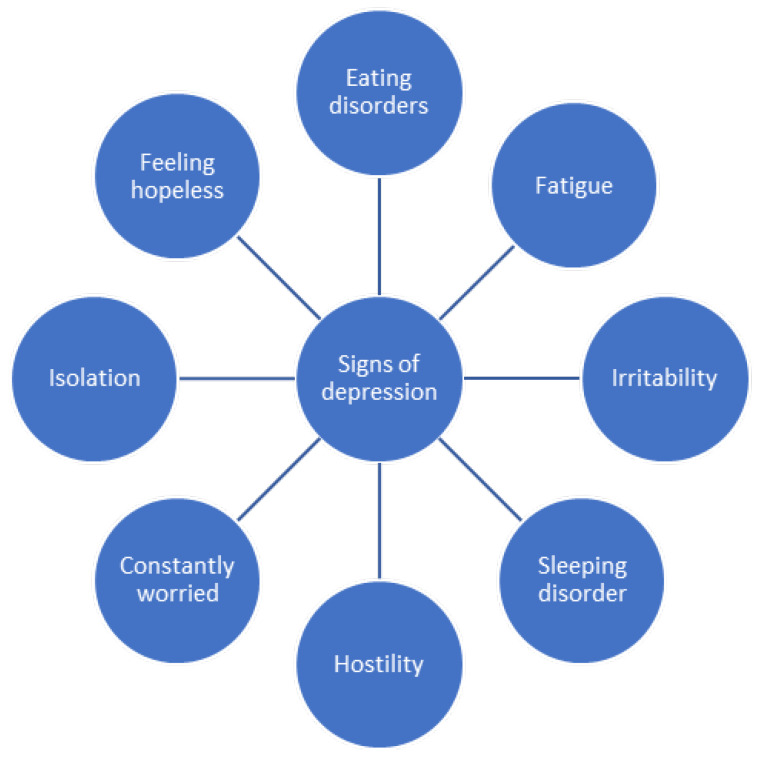
Signs of depression [87,88].

The early detection of symptoms suggesting depression is crucial, as screening and prevention are the key to a healthy individual with less medical conditions. The most common signs of depression are listed in Figure 4.

An important level of burnout is often associated with an increased risk of developing cardiovascular diseases like acute coronary syndrome and stroke [89]. Workers with higher burnout scores also showed ECG changes and higher LDL levels [90]. An increased level of stress can lead to an elevation of the systolic and diastolic blood pressure and increased heart rate, potentially evolving into a cardiovascular disorder. Migraines and ulcers are other possible negative outcomes [87,91].

Burnout in healthcare workers and the consecutive addictive habits or psychological disorders can also negatively impact patient care [92,93], with decreased patient satisfaction, with less compliance leading to prolonged recovery and hospital stay. Research covering 16 US hospitals proved increased patient satisfaction, better follow-up, and shorter hospital stays [94] by raising the job fulfilment of medical staff, with the eventual outcome being lower expenses for the medical system. Physicians pleased with their occupational achievements tend to offer competent care for patients in a shorter time, thus being perceived as prompt, efficient, and emphatic professionals [95]. Staff well-being positively impacts both patients and physicians [96]. When staff are well, they exhibit more patience in evaluating and explaining to patients. This leads to higher job satisfaction, lower stress levels, and a reduced likelihood of experiencing burnout. Raising costs for hospitals could originate in the need for training newly hired personnel, the frequency and length of medical leave, or the poor performance of employees, developing as a consequence of burnout in healthcare workers [97,98,99].

## 7. Discussion

The appearance of the first signs of burnout and its progression as a syndrome, affecting the three dimensions or contributing to different pathologies, can depend on a series of factors. The literature mentions behavioural factors [40,41,42] specific for different types of work environments emerging from neuropsychological overload (workload, accelerated work pace, high expectations regarding the final result, and others). Among the mentioned occupational risk factors [100] (work overload, professional experience), working the night shift [101] plays the most important role by far, being linked to the appearance of various pathologies due to sleep deprivation. This leads to disturbances in melatonin level [102], with lower levels being identified in patients with cancer and cardiovascular, metabolic, and psychological disorders. The non-occupational risk factors with a higher impact on the stress level are younger age, being in a relationship, and having proper social support [100]. The gender factor is yet to be elucidated; some authors [103] suggest that female medical personnel are more susceptible to burnout and stress in a shorter period compared to male medical personnel, while others have observed higher levels of burnout in older male healthcare workers [104].

Medical personnel [103] are even more prone to developing burnout because there is a constant demand for medical care, available around the clock, to improve the quality of a patient’s life. The urgency of having to make difficult decisions, the psychological burden, and prolonged work schedule are additional stressors for employees active in the health domain.

This situation was aggravated during the COVID-19 pandemic, which generated more stress with the introduction of new preventive measures, protocols, treatments, and vaccines. On top of that, the complexity of this pathology, the clinical severity of the cases, and the risk for contamination meant longer shifts, more pressure at the workplace, and a higher risk of developing burnout [105]. Healthcare workers thus perceived increased pressure at work, and the younger employees, with lower professional experience, reported a higher level of burnout than the older ones who had accumulated more experience in their professional life [105,106].

We identified responses associated with a high level of burnout: addictions like drug and alcohol abuse and depression with an increased incidence of suicide attempts among medical personnel [104].

Some authors have suggested physical activity as a protector against burnout [107]. It is efficient as a coping mechanism in dealing with job-related stress. Another protector, valuable also as self-confidence booster, is the social support from family and friends. Partners are perceived as a positive factor that increases the quality of life and relates to a lower level of burnout [108]. The preventive supplementation of melatonin could prove to be effective as a protector due to its effects in situations like sleep disorders, cognitive impairment [63], and even cancer therapy protocols [109].

The tools for diagnosing the burnout phenomenon are specific questionnaires. Our research identified multiple sources for questionnaires, some of them are applicable only to certain types of populations like the working and non-working, medical personnel, teachers, athletes, and students.

The most validated questionnaire in use for medical personnel is the MBI, with a specific version for healthcare workers. Some authors have tried to correlate the questionnaire’s outcomes with monitoring the serum levels of certain biomarker like S 100B [68].

Changes in blood levels of certain hormones like cortisol and metabolites have proven useful in evaluating prolonged exposure to stress, but variations due to circadian diurnal rhythms can occur; DHEA could prove useful but only in young subjects; prolactin can be useful in male subjects. The study on melatonin [61], its precursors, and its metabolites is of great scientific interest at the international level, being correlated with multiple pathological processes (cardiovascular, neurological, oncological diseases, important endocrinological, and metabolic alterations) [110]. The development of modern analysis techniques, from enzyme immunoassays (ELISA) to radioimmunoassay, chromatographic analysis coupled with mass spectrometry, type GC-MS or LC-MS, have allowed the separation and identification of melatonin, its precursors, and its metabolites in blood, urine, and saliva.

## 8. Conclusions

The burnout phenomenon is reflected in three dimensions: emotional exhaustion, depersonalization, and personal accomplishment. Its effects are most frequently evaluated with the Maslach Burnout Inventory questionnaire.

Identifying proper appraisal methods for the burnout syndrome should be performed depending on the type of subjects under evaluation. Observing the targeted population requires specific information about individual particularities, stress generators at the workplace, various risk factors (society, family), and biomarkers for stress. These remarks will, in turn, aid to provide prevention strategies to help control the burnout level of an individual on a collective level.

For healthcare workers, biohazard exposure is a significant risk, primarily from needle and sharp instrument injuries, resulting in substantial financial and time-management consequences. High demands in hospital settings increase burnout, depression, and suicidal ideation among these workers, reducing patient satisfaction and increasing hospital stays. At the same time, shift work, common in the medical profession, leads to cognitive impairment and changes in brain activity, contributing to burnout.

Protectors and coping mechanism are crucial in medical personnel, as improving job satisfaction among medical staff will lead to better patient outcomes and lower medical systems costs. The coping mechanisms indicated or applied can be very different and can also, unfortunately, generate negative behaviours, which might imply variable addictions. The same outcome can occur once the coping mechanisms are out of reach or have not been properly undertaken. According to our research, early signs of psychiatric disorder can appear.

We believe that the issue at hand mandates further research as we elaborate our own study protocol for evaluating the effects of burnout on exposed medical personnel, taking into consideration possible gender-related differences in the study group. Our upcoming research will apply questionnaires and biomarker monitoring.

## Figures and Tables

**Table 1:** Risk factors for developing burnout in medical personnel.

Occupational	Non-Occupational
Night shift	Younger age
Several workplaces	Marital status
Professional experience	Gender
Job satisfaction	Social support
Training	Having children
Work stress	Personality

**Table 2:** Instruments for evaluating burnout.

Questionnaire	Targeted Occupational Population	Evaluated Dimensions	Number of Items/ Languages Available	Details for Interpretation/ Scoring	Availability
Maslach Burnout Inventory [3,20]	Multiple	Three dimensions: Emotional exhaustion (EE) Depersonalization (DP) Low personal accomplishment (PA)	22 (MBI-HSS) 16 (MBI-GS)/EN and available translations for other languages	Low to high burnout evaluated for each dimension	Paid licence
Copenhagen Burnout Inventory [21,22]	Multiple	Three dimensions: Personal burnout Professional burnout Client-related burnout	19/EN, Danish	Low to severe burnout	Free
Oldenburg Burnout Inventory [23,24]	Multiple	Three dimensions: Physical exhaustion Mental exhaustion Disengagement from work	16/EN, (validated in China, Philippines, India, Brazil Portugal, Slovenia, Poland, Pakistan, Malays, Greece, Nigeria)	Low, moderate, or high; the higher the score, the higher the level of burnout	Free
Karasek Job Content Questionnaire [25]	Multiple	Three dimensions: Decision latitude Psychological demands Social support	49/EN, validated in 30 countries (amongst them are the following: Belgium, Bulgaria, France, Iceland, Iran, Italy, Japan, Malaysia, Romania, Spain, Thailand, Venezuela)	Measures the high-demand/low-control/low-support model of job strain development	Paid licence
Burnout Assessment Tool with 2 sections BAT-C and BAT-S [26]	Working and non-working population	BAT-C 4 dimensions: Exhaustion Mental distance Emotional impairment Cognitive impairment BAT-S 2 dimensions: Psychological component Psychosomatic component	Total 33 BAT-C 23 BAT-S 10/EN, Flemish, Dutch	No risk of burnout, at risk of burnout, very high risk of burnout, with statistical norms available for each dimension for the total level	Free
Burnout Clinical Subtypes Questionnaire [27]	Multiple	The frenetic subtype with 3 subscales: Ambition Overload Involvement The underchallenged subtype with 3 subscales: Indifference Lack of development Boredom The worn-out subtype with 3 subscales: Lack of acknowledgement Neglect Lack of control	36/EN, Latvian	Differentiates types of burnout depending on the level of dedication at work	Free
Questionnaire for the Evaluation of Burnout Syndrome at Work [28]	Multiple	Four dimensions: Enthusiasm for work Psychological exhaustion Indolence Guilt feeling	20/EN, Dutch, German, French, Italian, Brazilian Chinese, Brazilian, Finnish Hungarian, Japanese, Korean, Norwegian, Ukrainian, Polish, Portuguese, Romanian, Slovenian, Spanish, Turkish, Swedish, Russian, Greek, Latvian, Croatian, Lithuanian	Low scores on enthusiasm for work and high scores on psychological exhaustion, indolence, and guilt indicate high level of burnout	Paid licence
Brief Burnout Questionnaire Revised for Nursing Staff [29]	Nurses	Burnout as a process with its antecedents and consequences	21/EN	Higher motivation at work implies lower levels of burnout	Free

**Table 3:** Biomarkers linked with high levels of stress.

Biomarker	Identification	Origin	Prediction/ Confirmation Value
Melatonin [43,44,45,46,47,48,49,50]	Blood, Saliva, Hair	Pineal gland	Confirmation
Cortisol [38,51]	Blood, Saliva, Hair	Hypothalamus	Confirmation
CRH—corticotropin-releasing hormone [52,53]	Blood	Hypothalamus	Prediction
DHEA—dehydroepiandrosterone [38,54]	Blood	Hypothalamus	Prediction
Thyroid hormones [38,55]	Blood, Hair	Hypothalamus	Prediction
ACTH—adrenocorticotropic hormone [38,44,47]	Blood	Pituitary gland	Prediction
Prolactin [38,56]	Blood	Pituitary gland	Prediction
Serum S 100B [57]	Blood	Astrocytes and oligodendrocytes	Prediction
BDNF—brain-derived neurotrophic factor [58,59]	Blood	Endoplasmic reticulum from dense core vesicle localized in hippocampus, basal forebrain, and cortex	Prediction
TNF-α—tumour necrosis factor alpha [60]	Blood	Micro-inflammation	Prediction
IL—interleukin [52]	Blood	Micro-inflammation	Prediction
CRP-C reactive protein [52]	Blood	Micro-inflammation	Prediction

**Table 4:** Protectors against burnout.

Protectors against Burnout
grit
resilience
psychological flexibility
social support
good employer–employee relationships
good infrastructure
presence of employee wellness or mental health service
work–life balance
institutional leadership
having a religious background or belief

## Data Availability

Data are contained within the article.
